# Curvature-corrected retinal registration of diagnostic OCT with instrument-integrated OCT

**DOI:** 10.1038/s41598-025-28922-6

**Published:** 2025-11-28

**Authors:** Marius Briel, Ludwig Haide, Michael Meyling, Benjamin Saalfrank, Philipp Matten, Nicola Piccinelli, Gernot Kronreif, Maria Francesca Spadea, Eleonora Tagliabue, Franziska Mathis-Ullrich

**Affiliations:** 1 Carl Zeiss AG, Oberkochen, Germany; 2 Laboratory for Surgical Planning and Robotic Cognition (SPARC), Friedrich-Alexander-University Erlangen-Nürnberg, Erlangen, Germany; 3 Institute of Biomedical Engineering, Karlsruhe Institute of Technology, Karlsruhe, Germany; 4 Department of Engineering for Innovation Medicine, Università di Verona, Verona, Italy; 5 Austrian Center of Medical Innovation and Technology (ACMIT), Wiener Neustadt, Austria

**Keywords:** Medical image registration, Vitreoretinal surgery, Optical coherence tomography, Medical robotics, Diseases, Engineering, Medical research, Optics and photonics

## Abstract

Vitreoretinal surgery, requiring precise microscale tissue manipulation, is well-suited for robotic assistance. Image registration enhances surgeons’ visual perception by aligning high-resolution preoperative OCT images with the intraoperative environment, improving visibility of anatomical features not seen in microscope images. However, optical distortions from the cornea, lens, eye curvature, and scanning patterns challenge the use of diagnostic data in robotic navigation. This study introduces a novel technique for curvature-corrected retinal registration, integrating diagnostic OCT with instrument-integrated OCT. The pipeline comprises feature extraction, curvature correction, initial alignment, and fine registration. Experiments using an artificial model eye and ex vivo porcine eye validate the method. Curvature correction achieves accuracy comparable to existing methods, with deviations of 17 $\upmu$m for the model eye and 460 $\upmu$m for the porcine eye. Post-registration, the fiducial marker error reduces to 103 $\upmu$m for the model eye and 318 $\upmu$m for the porcine eye. Our method provides intraoperative diagnostic context, enabling reliable topological assistance in retinal robotic systems.

## Introduction

Epiretinal membranes (ERM) are a common macular condition, affecting up to 12 % of the population^1^. ERM peeling is a surgical intervention aimed at removing scar tissue from the retina to surgically repair vision. Preoperative optical coherence tomography (OCT) is extensively utilized in ophthalmology to detect and pinpoint ERM, as these transparent membranes are challenging to distinguish in fundus images. The peeling procedure involves creating a flap and meticulously removing the ERM from the retina using circular motions^2^. For retinal surgeons, the most delicate aspects of ERM peeling are flap creation and membrane grasping without causing excessive indentation of the retina, which could cause irreversible retinal trauma. Robot-assisted membrane grasping offers potential benefits by minimizing hand tremors and enabling precise tissue manipulation^3^.

Instrument-integrated optical coherence tomography (iiOCT) imaging is increasingly adopted to assist vitreoretinal surgeons in overcoming the limited depth perception inherent in surgical microscopes^4^. By integrating an optical fiber into microsurgical tools like picks and forceps, surgeons can measure the distance between the instrument’s tip and the retina, facilitating flap creation and peeling^5,6^. Previous research has shown that iiOCT combined with robotics can create a topographic map of the retina’s first layer^7^. However, the reduced image resolution of iiOCT poses challenges for distinguishing tissues beyond the retinal boundary layer. Optimal grasping points are generally characterized by minimal discontinuities between the ERM and the underlying retinal nerve fiber layer^8^.

High-resolution OCT volume scans of the perimacular region assist in identifying specific areas, such as these optimal grasping points. However, the disparate coordinate systems hinder the use of preoperative images for surgical navigation. Extensive research exists on mono-modal registration of retinal OCT data to enhance image quality, expand the field of view, and conduct longitudinal studies. Multi-modal registration has also been explored to fuse OCT images with other imaging techniques, such as fundus imaging^9^ and fluorescein angiography^10^. While Fleming et al. have examined the registration of preoperative OCT images with intraoperative microscope images to identify ERM edges for peeling initiation^11^, the registration of diagnostic OCT with iiOCT remains unexplored.

Volumetric transformation-based registration methods, which utilize intensity differences or cross-correlation, are commonly employed when retinal data is generated by the same imaging protocol. In contrast, image-features-based registration methods use distinct anatomical features of the retina to determine the necessary transformation, as discussed by Pan et al.^12^. These features include different landmark points, curves, surfaces, or combination thereof, and are effectively used for both mono- and multi-modal registration due to their lower computational complexity. Point-based approaches utilize image features such as the fovea, optic disc, and blood vessel bifurcations^12^. Layer-based methods involve segmenting retinal layers, with the inner limiting membrane (ILM) and retinal pigment epithelium (RPE) being commonly segmented layers. For instance, the OCTRexpert algorithm employs seven retinal layers for mono-modal registration, starting with ILM and the RPE alignment and incrementally incorporating additional layers, as described by Pan et al.^13^. Rivas-Villar et al. perform multi-device OCT registration of longitudinal scans by using both vessel bifurcations and layer information^14^. In multi-modal registration, it is common practice to convert both modalities into a unified representation^15^. Liu et al. generated point cloud data from endoscope-integrated OCT and micro-computed tomography (µCT). They utilized a convolutional neural network to extract features from both point clouds, performed rigid matching, and employed a neural deformation pyramid network for nonrigid refinement^16^.

Retinal imaging data is affected by distortions caused by the optical properties of the cornea and lens, as well as the curvature of the eyeball. Steidle and Straub developed an optical method to correct display distortions in posterior segment OCT images by estimating the shape of the human eye, involving three-dimensional ray tracing through OCT scan optics and ocular surfaces using optical simulation^17^. Their eye model allows for adjustments of parameters such as axial eye length and corneal curvature, with tolerance analysis revealing high sensitivity to axial length variations, as noted by Bumstead et al.^18^. Conversely, Kuo et al. employ both numerical and analytical models to reorient the A-scans in OCT images, validating corrected images against MRI^19^. A significant limitation of optical methods is the need for customization with biometric measurements from the measured eye, making curvature correction sensitive and error-prone. Grytz et al. introduced an empirical nonlinear distortion correction method that was validated using MRI images and implanted glass beads with known diameters, and applied to tree shrews^20^.

This study aims to bridge the gap between preoperative diagnostic 3D imaging and intraoperative robotic assistance by proposing a preoperative-intraoperative registration pipeline that includes curvature correction (CC) of extraocular OCT. Specifically, the contributions of this work are:

1. Generation of both preoperative and intraoperative retinal point clouds of an artificial eye phantom and a closed-sky ex vivo porcine eye. Source point clouds are obtained through segmentation of preoperative OCT volumes and feature extraction, while target point clouds are acquired through intraocular instrument movements by a robotic system while performing iiOCT distance measurements.
2. Proposal of a multi-step registration pipeline that takes source and target point clouds as input and generates the corresponding deformation field. This pipeline includes a novel CC method based on sphere fitting, leveraging the accurate intraocularly measured retinal curvature.
3. Evaluation of appropriate robotic iiOCT trajectories and required point cloud densities to achieve successful registration, along with assessment of the benefits of a supplemented CC step within the registration pipeline.
4. Discussion of accuracy and time requirements that make our method applicable within the clinical workflow of vitreoretinal surgery.

## Method

**Fig. 1:**
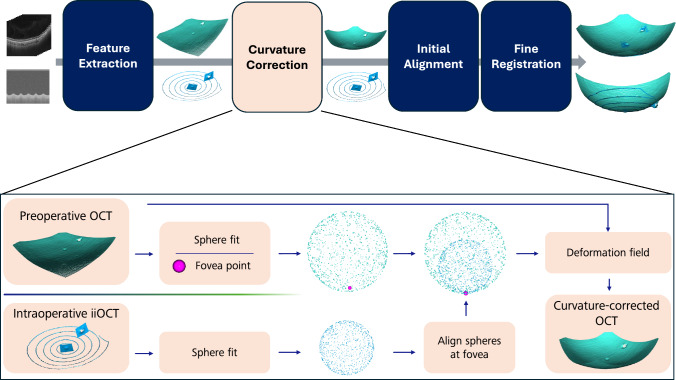
The proposed method for curvature-corrected registration integrates diagnostic OCT with instrument-integrated OCT. The CC stage utilizes the accurate curvature information provided by the iiOCT to rectify the curvature of the diagnostic OCT scan.

Our approach for curvature-corrected registration of diagnostic OCT with instrument-integrated OCT point clouds, as illustrated in Fig. 1, consists of the four key stages: *feature extraction*, *CC*, *initial alignment*, and *fine registration*.

The feature extraction stage is dedicated to extracting the ILM layer within the OCT data and converting it into a point cloud representation. The CC stage addresses any distortions present in the diagnostic OCT data, ensuring its geometric representation is accurate by leveraging iiOCT data. Lastly, initial alignment and fine registration stages work together to align the OCT data with the iiOCT data within the intraoperative robotic coordinate system.

### Feature extraction

Feature-based registration methods typically rely on retinal vasculature or retinal layer information as key features for registration^12^. However, detecting blood vessels in iiOCT poses a considerable challenge. Additionally, iiOCT scans can primarily be utilized to segment the retinal boundary layer and potentially the bright RPE. Beyond its vasculature and layers, the healthy human retina features two consistent landmarks: the fovea and the optic nerve head (ONH). These landmarks exhibit minimal variation and have a predictable relative orientation based on whether they belong to the left or right eye. Consequently, they are utilized for the preliminary alignment of source and target point clouds. Following this initial alignment, the extensive retinal boundary layer is employed for fine registration.

Feature extraction is carried out in two distinct stages. The first stage involves extracting the retinal surface from both modalities and represents it as point clouds. In the second stage, we manually extract additional point features from the generated point clouds.

To segment the diagnostic OCT volumes, the first white pixel encountered along the imaging direction of each one-dimensional A-scan within the three-dimensional C-scan is selected to represent the surface coordinate, thereby generating a 3D segmentation mask. In a similar manner, the iiOCT M-scan, which consists of temporally consecutive A-scans, is segmented to produce a 2D segmentation mask. To ensure registration accuracy, we manually annotated the retinal boundary layer using 3D Slicer 5. Numerous algorithms are available for OCT segmentation of C-scans, B-scans, and A-scans^21^. To transform the iiOCT boundary segmentation into 3D space, each segmented surface point is paired with its corresponding robot pose, given that the iiOCT probe is attached to a robot. The *i*-th point in the iiOCT retinal point cloud is determined as

1 $$\begin{aligned} \textbf{p}_i^{\text {iiOCT}} = \textbf{x}_i^{\text {iiOCT}} + d_i^{\text {iiOCT}} \cdot \dfrac{\textbf{v}_i^{\text {iiOCT}}}{\Vert \textbf{v}_i^{\text {iiOCT}}\Vert } \,, \end{aligned}$$

where $\textbf{x}_i^{\text {iiOCT}}\in \mathbb {R}^3$ and $\textbf{v}_i^{\text {iiOCT}}\in \mathbb {R}^3$ denote the position and orientation of the iiOCT probe tip in the robot base coordinate system, respectively, and $d_i^{\text {iiOCT}}\in \mathbb {R}_+$ represents the measured distance to the retina. Representing the two different modalities as point clouds leverages point cloud registration methods.

### Curvature correction

While the methods proposed by Steidle and Straub, and Kuo et al. necessitate information about refractive inidices and axial eye length^17,19^, the approach presented here operates independently of these measurements, by incorporating curvature information from iiOCT. The objective of this pipeline component is to transform the curvature-distorted OCT point cloud into a curvature-corrected OCT point cloud, ensuring its curvature aligns with that of the iiOCT point cloud.

Let $\{\textbf{p}_i^m \in \mathbb {R}^3 \}$ denote the point cloud of OCT modality *m*, which can be either diagnostic OCT or intraoperative iiOCT, within their respective coordinate systems. The iiOCT signal is free from optical distortions, which allows for a sphere fit to accurately represent the true radius of the retina, as evidenced by previous research^22^. A sphere is defined by its center $\textbf{c}\in \mathbb {R}^3$ and radius $r\in \mathbb {R}_+$, which minimize the sum of squared distances of the sample points from the sphere surface *S*:

2 $$\begin{aligned} S^m: \mathop {\mathrm {arg\,min}}\limits _{\textbf{c}^m, r^m} \sum _i \left( \Vert \textbf{p}_i^m - \textbf{c}^m\Vert - r^m \right) ^2 \,. \end{aligned}$$

Although the eye is not a perfect sphere, it can be adequately approximated as one for the purpose of rough CC in this step of the pipeline^23^. Since the fovea lies on the optical axis and distortions around the fovea are minimal, the two spheres are aligned in the fovea. In contrast, the distances between the spheres increase toward the periphery, where distortion effects become more pronounced.

To correct a specific distorted OCT point $p_i^{\text {OCT}}$, a line $L_i(t) = \textbf{p}_i^{\text {OCT}} + t(\textbf{c}^{\text {OCT}} - \textbf{p}_i^{\text {OCT}}), \, t \in \mathbb {R}$ is drawn from the point $p_i^{\text {OCT}}$ toward the center $\textbf{c}^{\text {OCT}}$ of the sphere fitted to the distorted OCT point cloud. Next, the intersections $\textbf{j}_i^{\text {OCT}}\in \mathbb {R}^3$ and $\textbf{j}_i^{\text {iiOCT}}\in \mathbb {R}^3$ between line $L_i$ and the lower hemispheres of the OCT sphere and the iiOCT sphere are computed. The differences $\varvec{\epsilon }_i = \textbf{j}_i^{\text {iiOCT}} - \textbf{j}_i^{\text {OCT}} \in \mathbb {R}^3$ between these intersections constitute the deformation field $\{\varvec{\epsilon }_i\}$. A point $\textbf{p}_i^{\text {OCT}}$ is corrected by adding the corresponding deformation vector:

3 $$\begin{aligned} \textbf{p}_i^{\text {OCT*}} = \textbf{p}_i^{\text {OCT}} + \varvec{\epsilon }_i \,. \end{aligned}$$

It is important to note that this approach does not project points onto spheres; rather, it preserves features present in the original point cloud through deformation.

### Initial alignment

The diagnostic OCT data is initially aligned with the iiOCT data within the robot’s coordinate system, ensuring that both point clouds are oriented to open upward. This alignment lays the groundwork for more precise fine registration.

First, the OCT point cloud is translated so that the fovea points coincide. Next, the imaging directions are aligned through a point cloud tilt correction. The two vectors $\textbf{n}^{\text {OCT}} = \textbf{c}^{\text {OCT}} - \textbf{p}_f^\text {OCT}$ and $\textbf{n}^{\text {iiOCT}} = \textbf{c}^{\text {iiOCT}} - \textbf{p}_f^\text {iiOCT}$, which point from the foveas to their respective sphere centers, are aligned using Rodrigues’ rotation formula. Lastly, the OCT point cloud $\{\textbf{p}_i^\text {OCT}\}$ is rotated around the *z*-axis, which represents the visual axis, as follows:

4 $$\begin{aligned} \textbf{p}_i^{\text {OCT}^\prime} = R_z(\phi ) \cdot \textbf{p}_i^{\text {OCT}}\,. \end{aligned}$$

This rotation aligns the ONHs $\textbf{p}_o^\text {OCT}$ and $\textbf{p}_o^\text {iiOCT}$, where $R_z(\phi )$ is the rotation matrix around the z-axis and

5 $$\begin{aligned} \phi = \arccos \left( \frac{\textbf{p}_o^\text {OCT} \cdot \textbf{p}_o^\text {iiOCT}}{\Vert \textbf{p}_o^\text {OCT}\Vert \Vert \textbf{p}_o^\text {iiOCT}\Vert }\right) \end{aligned}$$

is the angle between the ONHs. This rotation, restricted to the *x*-*y* plane, is intended to prevent the introduction of a tilt, as the distance in *z*-direction between the ONHs can be significant, even after CC. General methods, such as random sample consensus (RANSAC), may fail during coarse registration due to the lack of distinctive features and the differences in curvature.

### Fine registration

Iterative closest point (ICP) is a widely used algorithm for aligning 3D point clouds given an initial guess of the rigid transformation^24^. In this work, ICP is employed in combination with CC, enabling the OCT point cloud to be first deformed and subsequently transformed to achieve the best rigid alignment with the iiOCT point cloud. Point-to-plane ICP is a variant of ICP that utilizes the normal vectors of the target points to minimize the distances between source and target clouds, demonstrating faster convergence compared to the original point-to-point version. ICP establishes correspondences between points ${\textbf {a}}$ of the source point cloud *A* and points ${\textbf {b}}$ of the target point cloud *B* using a closest point criterion, which seeks to find the closest point in the target cloud for each point of the source cloud. If the distance between points is below a certain threshold, a correspondence is established, and the pair is added to the set of corresponding points $K=\{({\textbf {a}},{\textbf {b}})\}$. Next, the transformation $(R,{\textbf {t}})$ that minimizes the error metric

6 $$\begin{aligned} E(R,{\textbf {t}})=\sum _{({\textbf {a}},{\textbf {b}})\in K}(({\textbf {b}}-(R{\textbf {a}}+{\textbf {t}}))\cdot \textbf{n}_b)^2 \end{aligned}$$

is computed, where ${\textbf {n}}_b$ is the normal of the respective target point *b*. ICP is terminated when either a maximum number of iterations, set to $j=100$, is reached or when an experimentally defined relative root mean square error of $10^{-6}$ is achieved. For this work, a correspondence distance threshold of 20 $\upmu$m is used.

Additionally, we explore whether the nonlinear transformations computed by nonrigid coherent point drift (CPD) can serve as an alternative to CC. Furthermore, given the time differences between diagnosis and surgery, non-rigid methods may be advantageous even in the absence of curvature distortions, as they can accomodate changes in intraocular pressure or intraoperative lesions. CPD models the points $a \in A$ as a Gaussian mixture model (GMM), meaning that each point *a* is represented as a Gaussian kernel, while the target points $b \in B$ are treated as data points^25^. The method maximizes the log-likelihood of the data points belonging to the GMM. Additionally, the deformation field is regularized to prevent extreme deformations and to ensure a smooth transformation field. Given that nonrigid CPD is computationally demanding, the OCT source point cloud is downsampled to a spacing of 300 $\upmu$m. When applying the deformation to the dense source point cloud, the four nearest neighbors in the downsampled cloud are identified using a k-d tree search. Subsequently, the corresponding four translation vectors are weighted and averaged.

## Experimental validation

**Fig. 2:**
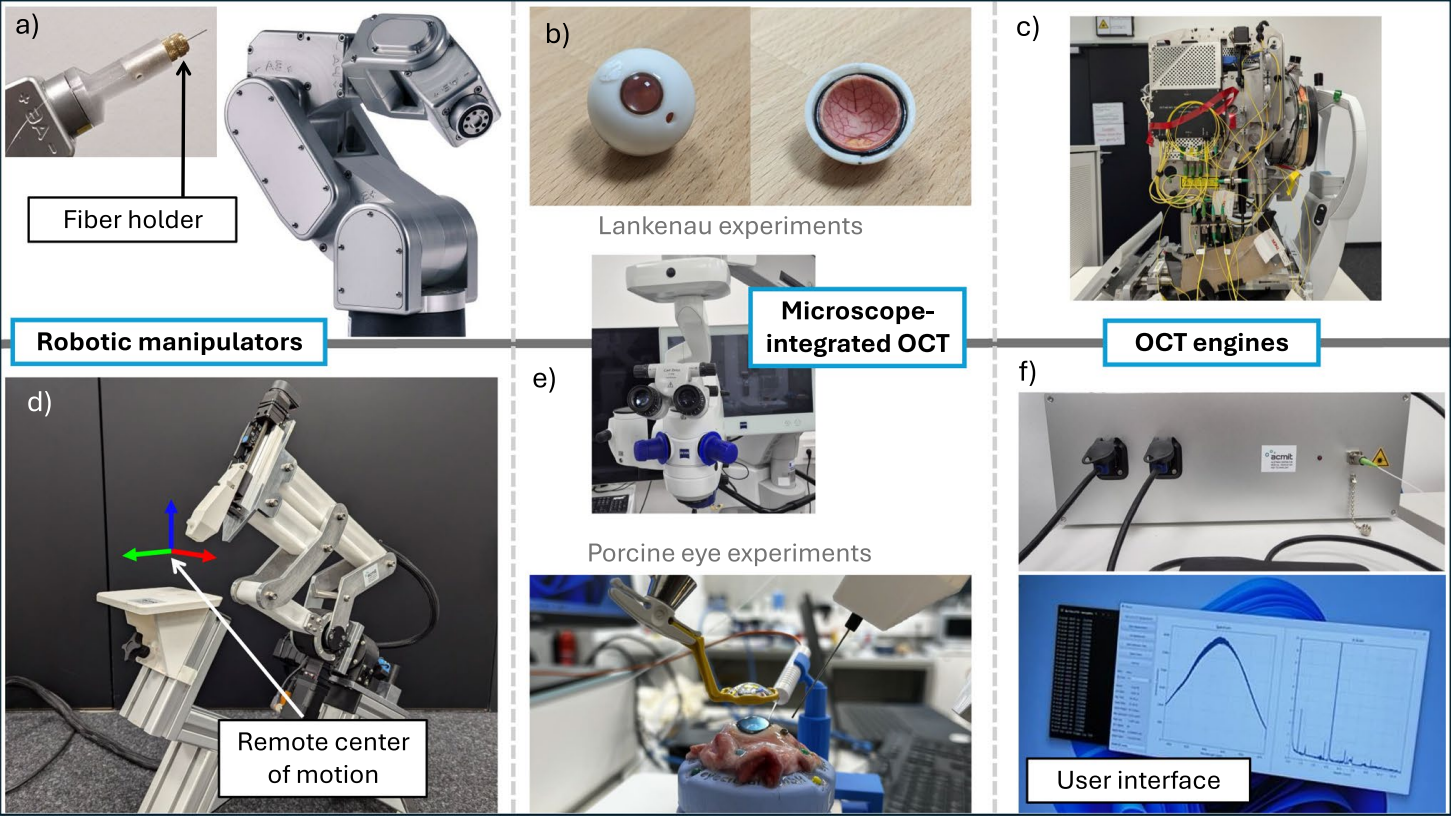
The ARTEVO 800 microscope-integrated OCT system captures diagnostic OCT volumes. (**a**) The Meca500 industrial robot is used for experiments involving the (**b**) artificial Lankenau eye phantom. (**c**) The modified IOL Master 700 provides real-time, dual-path OCT measurements. (**d**) The GEYEDANCE robotic manipulator, specifically designed for vitreoretinal surgery, is used for (**e**) porcine eye experiments. (**f**) A custom-built OCT engine is utilized for common-path iiOCT sensing.

Experiments validate the method using an artificial model eye and an ex vivo porcine eye, utilizing two distinct experimental setups for specific validation scenarios (Fig. 2). The GEYEDANCE surgical system, designed for vitreoretinal surgery, facilitates experiments in realistic surgical conditions, while the industrial robot offers enhanced precision.

### Data acquisition

Two different test eyes are employed to evaluate the proposed registration pipeline. The Lankenau model (*Modell-Augen Manufaktur Dr. Eva Lankenau*) is a model eye with realistic geometry specifically designed for OCT recordings. The anterior half of the model eye consists of a cornea and lens made of silicone. The posterior half contains the retina, which includes anatomical features such as blood vessels, the foveal pit, the ONH, and multiple retinal layers. The two halves can be assembled and filled with water to facilitate realistic closed-sky scanning. The model exhibits geometry, refractive power, and retinal structure that closely resemble those of a healthy and rigid human eye. Conversely, porcine eyes are frequently used as surrogates for human eyes in research due to their availability and similar anatomy, which includes the cornea, lens, retinal layers, and ONH. A significant difference, however, is the absence of a macula and fovea^26^. Additionally, ex vivo eyes often exhibit post-mortem mobile retinal detachments, which complicate accurate registration. The porcine eyes were sourced from a local butcher. Under closed-sky conditions, the anterior part of the eye, including the lens and cornea, is left intact to preserve the natural barriers. To prevent the cornea from drying out, a viscoelastic agent is applied. In contrast, open-sky conditions, which involve the removal of the anterior part, are frequently observed in research to avoid imaging distortions and to allow direct access to the vitreous and retina.

In this work, two distinct experimental setups are utilized, each specifically designed for either open-sky and closed-sky data acquisition (Fig. 2). For experiments involving an artificial model eye, the robotic manipulator used is the Meca500^®^ (*Mecademic Robotics, Montreal, Canada*), a six-arm industrial robot with a position repeatability of 5 $\upmu$m. Originally developed as a Fourier-domain OCT for biometry, the IOLMaster 700^®^ (*Carl Zeiss Meditec AG, Jena, Germany*) is adapted for iiOCT sensing in this study. Within the open-sky Lankenau eye, the end-effector tip is maneuvered over a 10 mm $\times$ 10 mm grid, with a spacing between points of approximately 50 $\upmu$m. The 2D grid is projected onto a sphere with a radius of 10 mm, which closely matches the radius of the Lankenau model. This projection ensures that the fiber tip positions remain close to the retinal surface throughout the entire recording, thereby maintaining sufficient signal strength.

For the ex vivo tests, the GEYEDANCE surgical system^27^, specifically designed for intraocular surgery, is employed. It consits of a customized 4-DoF robotic manipulator with a hardware-defined remote center of motion (RCM) and a high-resolution fiber-based OCT setup. The GEYEDANCE OCT system features a working distance of 12.8 mm and an A-scan depth of 1024 pixels, resulting in a resolution of 12.5 $\upmu$m. While the sampling rate is adaptable, an update rate of 50 Hz is used in this study. As for the Lankenau experiment, a 10 mm $\times$ 10 mm end-effector grid with a spacing of approximately 50 $\upmu$m is implemented. This trajectory is executed under closed-sky conditions with the RCM constraint, as would be typical in realistic clinical settings. The porcine eye data corresponds to the reconstructed porcine eye point cloud used in our modeling work^7^, which focused on modeling the local retinal geometry.

Closed-sky C-scans are acquired with the ZEISS ARTEVO 800 RESCAN^®^ (*Carl Zeiss Meditec AG, Jena, Germany*) microscope-integrated OCT, which serves as a substitute for a diagnostic OCT device. The scan field is adjusted to coincide with the scanned area of the iiOCT, consisting of $512 \times 128$ A-scans, each comprising 1024 pixels, with an imaging depth of 2.9 mm in tissue. This choice of imaging depth results in cut-off corners in the Lankenau experiment but enhances the resolution $\delta z$. The scan field is determined by counting the number of voxels between fiducial markers of known distance, as the nominal scan field is only applicable for flat surfaces. The ZEISS ARTEVO system’s model scales the point cloud based on the entry angle; however, microscope-integrated OCT faces challenges due to unknown factors, such as the inaccurate optical path, including the wide field lens^28^. Alternatively, feature points, such as the fovea and ONH, could be utilized to match the fovea-ONH-distances in both point clouds. The resulting voxel dimensions are $\delta x_L$ = 24.4 $\upmu$m, $\delta x_p$ =31.3 $\upmu$m, $\delta y_L$ = 97.6 $\upmu$m, and $\delta y_p$ = 125 $\upmu$m for the lateral dimensions, with an axial dimension of $\delta z$ = 2.8 $\upmu$m. The lateral voxel dimensions of porcine eye scan are increased compared to the the Lankenau model due to the larger scan field.

A Dell laptop with Windows 11, equipped with a 12th Gen Intel(R) Core(TM) i5-1245U 1.60 GHz processor and 16GB of RAM, serves as the computing platform used to control the robot and execute the registration algorithms programmed in Python.

### Data processing

Various robotic trajectories are assessed to determine the extent to which data acquisition can be minimized - thereby saving valuable operating room time - without compromising registration performance. Additionally, different spacings between scans are evaluated to explore the effects of increased instrument tip velocities. For a fixed iiOCT frequency, higher velocities result in larger spacings. In total, two distinct trajectory types, each with three different point spacings, are synthetically derived from dense grid scans with a spacing of approximately 50 $\upmu$m. This approach ensures highly resolved fiducial markers for all robot trajectories and enhances comparability.

**Fig. 3:**
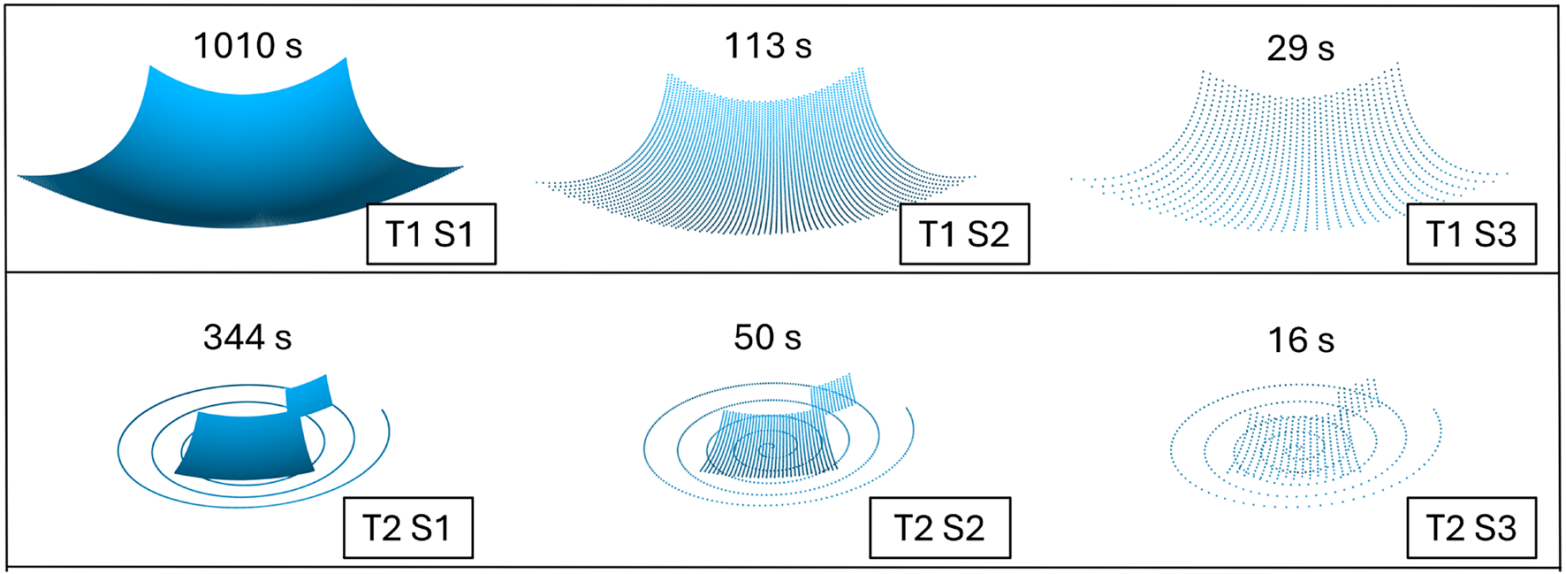
The combinations of trajectory (grid, spiral plus feature patches) and point spacing (50 $\upmu$m, 150 $\upmu$m, 300 $\upmu$m) yield six distinct configurations.

Trajectory T1 is the 10 mm $\times$ 10 mm grid pattern that covers the fovea at its center and the ONH in the periphery. Trajectory T2 is a 5 mm $\times$ 5 mm grid pattern that also centers on the fovea. Additionally, a 2 mm $\times$ 2 mm grid pattern is centered at the ONH location. An additional spiral trajectory starts at the center above the fovea and extends with 5 coils to a final radius of 5 mm. The different spacings - S1, S2, and S3 - have intervals of approximately 50 $\upmu$m, 150 $\upmu$m, and 300 $\upmu$m, between consecutive spiral points, respectively, and are obtained through subsampling. In total, the combinations of trajectory and point spacing result in six combinations (Fig. 3).

Given a robotic velocity of $v =$ 2 mms and a scanning frequency of 40 Hz for S1, the resulting spacing is 50 $\upmu$m. Under these conditions, the acquisition times are 344 s for T2 and 1010 s for T1. By increasing the spacing, it is possible to achieve higher speeds, which reduces the acquisition times to 16 s for T2 and 29 s for T1.

### Validation method

In similar studies, the positions of anatomical landmarks in both modalities are compared after registration to evaluate registration accuracy. However, the fovea and ONH are the only visible landmarks in the iiOCT and are utilized for registration. Therefore, fiducial markers are placed in the test eyes to provide additional validation points. The fiducial markers are not used for the registration process. An alternative approach is the calibration approach by Zhou et al., which involves localizing the instrument tip within the OCT volume and subsequently determining the transformation between the coordinate system of the microscope-integrated OCT and the robot’s coordinate system^29^. However, this validation would not be applicable if a preoperative OCT device were used.

**Fig. 4:**
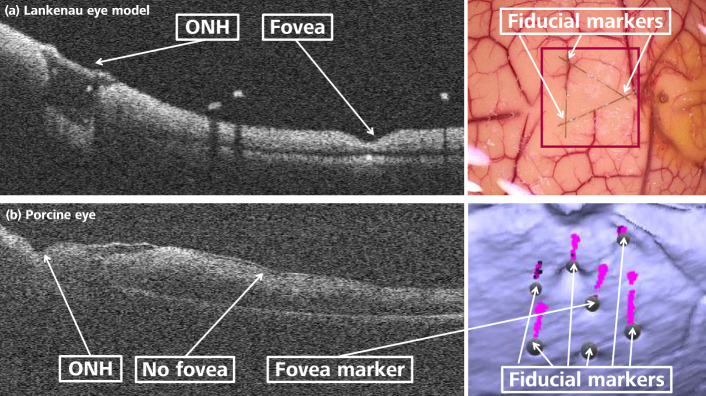
(**a**) The Lankenau eye model features geometric landmarks such as the fovea and the ONH, which are visible in the B-scan. This model allows for the placement of a triangular arrangement of 5 mm fiducial marker wires on the retina. (**b**) An intact porcine eye lacks a fovea and accessibility, necessitating the use of stitched wires as markers. The image displays fiducial markers alongside the segmented porcine eye iiOCT point cloud.

The fiducial markers in the Lankenau eye consist of three tungsten wires, each 50 $\upmu$m thick and 5 mm long, which are clearly visible in both modalities. These wires are arranged in a triangular formation within the macular area, which is the region of interest for ERM peeling (see Fig. 4a). The intersections of the three wires, forming the corners of the triangle, define the validation points.

In the porcine eye, seven wires, each with a thickness of 100 $\upmu$m and a length of 2 mm, are stitched toward the intraocular space from the back of the eye. This is illustrated in Fig. 4b, where retinal points are colored blue and wire points are colored magenta. The central wire serves as a substitute for the fovea, which is absent in porcine eyes but essential for our registration pipeline.

The mean fiducial marker error (MFME) serves as an indicator of how accurately a specific point, preoperatively planned in the diagnostic OCT point cloud, can be located within the robot’s coordinate system. This metric enables the evaluation, whether the necessary accuracy is achieved for automated grasping at preoperatively planned grasping points. The MFME is expressed as follows:

7 $$\begin{aligned} \text {MFME} = \frac{1}{N} \sum _{i=1}^{N} \sqrt{(x_i^{\text {iiOCT}} - x_i^{\text {OCT}})^2 + (y_i^{\text {iiOCT}} - y_i^{\text {OCT}})^2 + (z_i^{\text {iiOCT}} - z_i^{\text {OCT}})^2} \,, \end{aligned}$$

where *N* is the number of fiducial marker points, and $(x_i^{\text {iiOCT}}, y_i^{\text {iiOCT}}, z_i^{\text {iiOCT}})$ and $(x_i^{\text {OCT}}, y_i^{\text {OCT}}, z_i^{\text {OCT}})$ represent the coordinates of the fiducial markers in the iiOCT point cloud and in the OCT point cloud after registration, respectively.

The Chamfer distance (CD) is particularly useful for assessing the overall alignment quality between two point clouds, as it considers the distribution of all points rather than just specific landmarks. This metric provides a global evaluation, extending beyond specific areas such as the macula, where the fiducial marker are located. Moreover, precisely locating the fiducial marker can be challenging and may introduce errors in the MFME. In contrast, the CD is independent of such annotations, making it a robust measure. The CD calculates the average distances from each point in one point cloud to its nearest neighbor in the other point cloud, and vice versa. It is calculated as follows:

8 $$\begin{aligned} \text {CD} = \frac{1}{2|P_{\text {OCT}}|} \sum _{\textbf{p}_{\text {OCT}} \in P_{\text {OCT}}} \min _{\textbf{p}_{\text {iiOCT}} \in P_{\text {iiOCT}}} \Vert \textbf{p}_{\text {OCT}} - \textbf{p}_{\text {iiOCT}}\Vert + \frac{1}{2|P_{\text {iiOCT}}|} \sum _{\textbf{p}_{\text {iiOCT}} \in P_{\text {iiOCT}}} \min _{\textbf{p}_{\text {OCT}} \in P_{\text {OCT}}} \Vert \textbf{p}_{\text {iiOCT}} - \textbf{p}_{\text {OCT}}\Vert \,, \end{aligned}$$

where $P_{\text {OCT}}$ is the set of points in the OCT point cloud and $P_{\text {iiOCT}}$ the set of points in the iiOCT point cloud. If, in certain areas, the point clouds do not overlap, these regions can distort the CD, resulting in outcomes that appear worse than they actually are. This issue is mitigated by cropping the point clouds to a *z*-axis aligned cylinder of maximum radius, ensuring complete overlap.

## Results

For the Lankenau model eye, the radius of curvature determined via iiOCT is 9.356 mm, which is consistent with the manufacturer’s specification of approximately 10 mm. In contrast, the radius obtained using standard OCT prior to correction is 20.6 mm, while post-correction, it measures 9.373 mm. The absolute difference between the iiOCT curvature and the corrected OCT curvature is minimal, at 0.017 mm. In the case of the porcine eye, the radius of curvature measured using iiOCT is 10.706 mm. The OCT measurement before correction is considerably higher, at 28.8 mm, whereas the radius after correction is 11.166 mm. The absolute difference between the iiOCT radius and the corrected OCT radius for the porcine eye is larger, at 0.460 mm, indicating that the correction is less effective compared to the Lankenau model eye. Overall, these findings highlight the effectiveness in improving the accuracy of OCT measurements, as curvature errors are maintained within the sub-millimeter range.

**Fig. 5:**
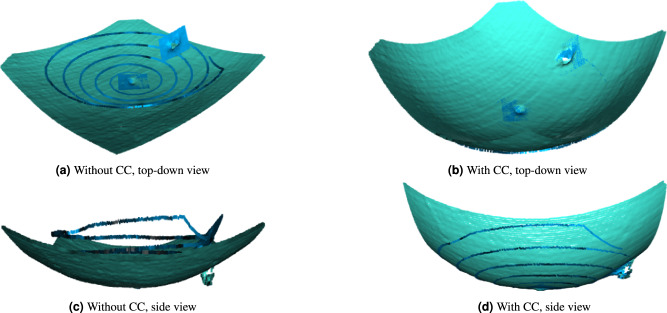
Qualitative registration performance on the Lankenau model data with and without CC. Mint color represents the diagnostic OCT data, while blue color represents the iiOCT data.

Figure 5 illustrates the qualitative registration performance on the Lankenau model data. In comparison to T2, the trajectory length is further reduced by decreasing the foveal patch size to a square with a side length of 2 mm. Within the macular region, the registration is successful for both curvature-corrected and curvature-uncorrected point clouds, as illustrated in Fig. 5a and b. However, in peripheral regions, the absence of CC results in a gap between the source and target point clouds. This discrepancy is particularly noticeable in the side view, as depicted in Fig. 5c.

Moreover, the registered point clouds depicted in Fig. 5d demonstrate that our CC method effectively preserves retinal features, including the fovea and the ONH, while simultaneously correcting the curvature.

**Fig. 6:**
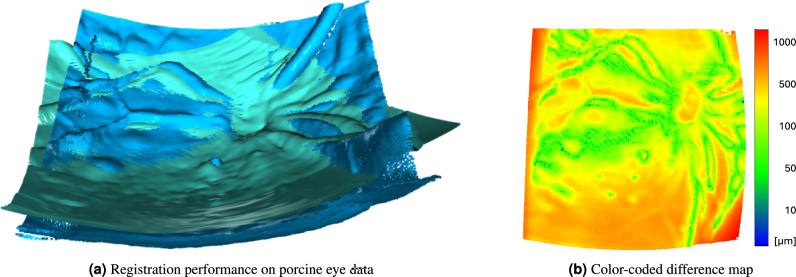
(**a**) Source (diagnostic OCT in mint color) and target (iiOCT T1 S1 in blue color) porcine eye point clouds after undergoing curvature-corrected registration. (b) Color-coded difference map illustrating the disparities between the OCT point cloud and the iiOCT point cloud following curvature-corrected registration.

Figure 6a presents the qualitative registration performance on the porcine eye using the combination T1 S1. Overall, the figure shows a good alignment of the point clouds, including at bumps, vessels, and the optic disc.

The color-coded heatmap in Fig. 6b demonstrates an overall good alignment, with the majority of distances being less than 1 mm. In the central area, most points are green, signifying a difference from the target point cloud of less than 100 $\upmu$m. The occasional blue points represent intersections between source and target point clouds. It is noteworthy that the error in the ONH located in the upper right corner is relatively high. The heatmap displays dark red hues in the top left and bottom right areas, which are solely attributed to the absence of iiOCT scanning in those regions, thereby justifying the cropping prior to calculating the CD. The bottom left section of the heatmap clearly indicates suboptimal registration results, characterized by an axial offset between the source and target point clouds.

**Fig. 7:**
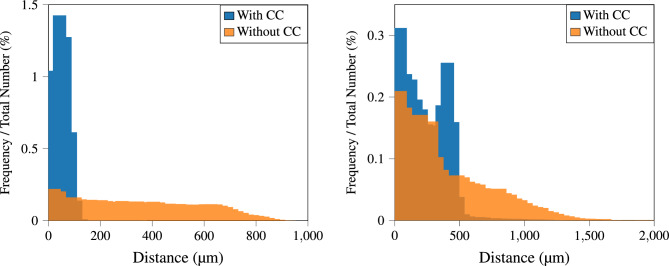
Comparison of distance distributions for T1 S1 presented for both the Lankenau model (left) and porcine eye data (right), each analyzed with and without CC.

Figure 7 illustrates the distribution of the distances that constitute the CD. For each point in the two point clouds, the nearest point in the other point cloud is identified, and the corresponding distance is calculated. The histograms on the left display the distance density distributions for the Lankenau model, while the distributions on the right pertain to the porcine eye, each with and without CC. For the Lankenau eye, the histogram associated with uncorrected registration reveals a high variance in distances, whereas the CC effectively concentrates the distance distribution to values below 150 $\upmu$m. A similar effect is observed with the porcine eye, albeit less pronounced. For curvature-corrected registration on the ex vivo tissue, very few distances exceed 0.5 mm, while without CC, the distribution extends up to 1.5 mm.

We conducted paired per-point comparisons of these minimum distances both before (baseline) and after applying our CC to test our hypothesis that CC enhances registration accuracy. For the Lankenau eye, the mean difference of 443 $\upmu$m with a standard deviation of 317 $\upmu$m from these comparisons indicates a clear improvement. The p-value, $p < 10^{-18}$, from the paired t-test confirms strong statistical significance. This improvement is also evident in the porcine eye, where the mean enhancement is 697 $\upmu$m with a standard deviation of 761 $\upmu$m.

Table 1 offers a comprehensive overview of the registration results acquired for both the Lankenau eye and biological tissue, assessed with and without CC.

**Table 1:** Quantitative registration performance, measured by MFME and CD, is evaluated for various trajectories and subsampling configurations on both the Lankenau model and porcine eye data, with and without CC.

Metrics	Lankenau model eye						Porcine eye					
	Without CC		With CC		Error reduction		Without CC		With CC		Error reduction	
	MFME [$\upmu$m]	CD [$\upmu$m]	MFME [$\upmu$m]	CD [$\upmu$m]	MFME [%]	CD [%]	MFME [$\upmu$m]	CD [$\upmu$m]	MFME [$\upmu$m]	CD [$\upmu$m]	MFME [%]	CD [%]
T1 S1	**134**	357	105	58.9	-21.6	-83.5	**318**	**395**	**320**	243	+0.63	-38.5
T1 S2	138	363	118	59.1	-14.5	-83.7	333	400	339	**234**	+1.80	-41.5
T1 S3	9100	1630	146	84.9	-98.4	-94.8	361	407	323	236	-10.5	-42.0
T2 S1	185	356	**103**	54.7	-44.3	-84.6	352	408	356	359	+1.42	-12.0
T2 S2	2340	**333**	178	89.0	-92.4	-73.3	360	396	350	263	-2.78	-33.6
T2 S3	209	371	123	**53.3**	-41.2	-85.6	333	397	335	256	+0.60	-35.5

Bold numerals are used to indicate which of the six configurations achieved the minimal error.

Generally, the registration results show improvement with an increase in trajectory length (from T2 to T1) and with a greater number of data points (transitioning from S3 to S1). Nevertheless, the registration performance remains relatively stable across the different iiOCT input data, indicating the potential to further reduce data acquisition times without compromising accuracy. While the MFME exhibits a slight but consistent improvement with CC for the Lankenau experiment, the CD shows an error reduction from 333 $\upmu$m in the best case without CC to below 89 $\upmu$m for all cases with CC. An MFME of 103 $\upmu$m and a CD of 54.7 $\upmu$m is achieved for T2 S1, representing an improvement of 82 $\upmu$m and 301 $\upmu$m, respectively. Moreover, CC stabilizes the registration performance, preventing divergences as large as 9.1 mm during optimization, as observed for T1 S3. For T2 S2, the overall alignment of the point clouds is good, but the large MFME indicates an incorrect rotation around the *z*-axis. The optimizer converged to a local minimum that produces a worse MFME in comparison to the initial alignment that preceded it.

In the porcine eye experiments, the MFME is not consistently affected by CC. Although the MFME increases slightly in four out of six configurations, for T1 S3, it decreased by 11 % from 361 $\upmu$m to 323 $\upmu$m. Conversely, the CD consistently demonstrates an error reduction of 135 $\upmu$m on average, equating to 33.8 %. The porcine eye registration achieves an MFME of 320 $\upmu$m and a CD of 243 $\upmu$m for T1S1.

**Fig. 8:**
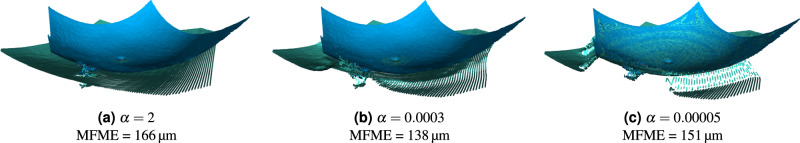
Registration results for nonrigid CPD with a varied coherence parameter $\alpha$ for the Lankenau model eye, along with the corresponding MFME, are presented. Nonrigidity is increased from left to right.

To evaluate whether nonrigid CPD could potentially replace CC, experiments with varying levels of nonrigidity are conducted. The parameter $\alpha$, which dictates the coherence of nonrigid CPD, is systematically adjusted. A higher $\alpha$ results in greater coherence and increased rigidity, whereas a lower alpha leads to reduced coherence and increased nonrigidity. The impact of this parameter sweep is illustrated in Fig. 8.

With a relatively large coherence of $\alpha = 2$, nonrigid CPD is unable to modify the curvature of the OCT point cloud effectively, as illustrated in Fig. 8a. As nonrigidity increases, i.e., with smaller $\alpha$ values, the overall alignment improves because CPD is afforded more flexibility. However, a small coherence parameter of $\alpha = 0.00005$ introduces discontinuities in the registered OCT point cloud, as OCT points remain uncorrected when no iiOCT points are nearby. This issue is particularly visible at the corners of the OCT point cloud shown in Fig. 8c. As shown in Fig. 8b, in the Lankenau experiment, a coherence parameter of $\alpha = 0.0003$ represents the optimal trade-off between alignment and fidelity. This parameter setting balances between the flexibility required for effective nonrigid registration and the preservation of the structural integrity of the point cloud. For the porcine eye, setting $\alpha =0.00001$ produces the optimal results, achieving an MFME of 748 $\upmu$m and a CD of 245 $\upmu$m. Even with the optimized $\alpha$ value, our proposed curvature-corrected rigid registration delivers superior results compared to non-rigid registration with CPD.

The mean computation time for ICP varies from 1.54 s for T2 S3 to 5.29 s for T1 S1. In contrast, the mean computation time for nonrigid CPD ranges from 7.90 s for T2 S2 to 63.20 s for T1 S1. The time required to compute the registration is negligible when compared to the duration of up to 17 min needed for robotic data acquisition.

## Discussion

### Curvature correction

The disparity in curvatures observed between OCT and iiOCT primarily arises from the scanning geometry utilized in OCT. Conversely, the C-scan image is generated by aligning A-scans in parallel. This method introduces a misalignment between the actual scan geometry and the representation displayed, which contributes to a flattened appearance of the retina^17^.

The curvature-corrected point clouds derived from both the Lankenau model and the porcine eye offer a more authentic depiction of the retina’s true anatomical structure. The greater variance between the target and actual radius observed in the porcine eye, post-correction, is likely due to its intricate anatomical features, deviation from a spherical form, and the less precise experimental setup. In the case of the Lankenau model, the least squares residuals from the sphere fitting measure 0.21 mm, whereas the porcine eye displays least squares residuals of 1.82 mm. Considering that the retina’s shape is not ideally spherical, the correction method could be refined by employing alternative fitting methods, such as ellipsoidal fitting. Nonetheless, when compared to advanced methodologies, such as the technique developed by Kuo et al.^19^, our CC technique exhibits competitive performance.

### Registration

The accuracy attained by our ICP-based curvature-corrected registration pipeline, measured at 103 $\upmu$m, is noteworthy. This level of precision could be adequate for addressing the use case of grasping at preoperatively defined grasping points. Given that the lateral spacing between iiOCT scans is approximately 50 $\upmu$m, this indicates that the achieved accuracy falls within the range of two voxels. The registration accuracy in the Lankenau model surpasses that of the porcine eye, likely due to the controlled experimental conditions. In the Lankenau model, the iiOCT data acquisition was conducted in an open-sky setup, where the robotic system operates without mechanical attachment to the eye. In contrast, the porcine eye experiment is conducted in a closed-sky configuration, where even minor deviations of the RCM could adversely impact the accuracy of data recordings. Furthermore, the signal quality in the porcine eye data is lower, as illustrated in Fig. 4. The reduced signal quality of the iiOCT, resulting from the beam divergence, is further affected by the medium through which the light waves propagate. In the Lankenau model, the medium is air, while in the porcine eye, the medium is vitreous humor, which exhibits greater attenuation compared to air. Additionally, obtaining high-quality OCT scans from ex vivo porcine eyes presents challenges due to the rapid degradation of the lens and cornea, which can lead to cloudiness.

The suboptimal performance of nonrigid CPD, which can result in incorrect curvature or discontinuities (see Fig. 8), could be enhanced by incorporating constraints within the expectation-maximization framework, specifically by penalizing lateral displacement. This approach would favor deformation in the *z*-direction to correct curvature while preserving a high level of coherence. Moreover, advancements in hardware could facilitate novel registration techniques. A higher-quality iiOCT system or OCT angiography could potentially capture vessel information, offering crucial features for improved registration. Additionally, integrating segmentation of the RPE layer could be advantageous. Existing implementations of colored ICP could leverage distinct labeling of the retinal surface and RPE to enhance registration accuracy.

For successful initial alignment, it is essential that both the fovea and the ONH are present in the recording, which is typically achievable in central OCT scans with a width of exceeding 10 mm. The inaccuracies observed, particularly in the porcine eye experiment, may stem from errors in the annotation process. Variability in pinpointing the center of the ONH, which has an approximate diameter of 2 mm, could affect registration accuracy. This issue could be mitigated by annotating the optic disc boundary rather than selecting a single point. Additionally, automating the feature extraction step could help eliminate human errors^30^. In scenarios where the foveal pit is absent due to disease, an alternative landmark, such as a macular hole, could be utilized for alignment.

### Validation method

The main method we use to evaluate the registration pipeline is the MFME, which is specifically designed to assess accuracy within the region of interest. This metric evaluates three or six distinct locations that represent points of interest, such as those where an ERM flap might be created. A limitation of this approach is the manual selection of fiducial marker points from the set of measured points, which means that the evaluation can be influenced by the resolution of the scans. In future work, this error could be substantially reduced by estimating wire crossovers instead of selecting actual points. Assessing registration accuracy in a closed-sky scenario presents particular challenges. Improving the resolution of the OCT system and reducing wire thickness could significantly enhance the results. An alternative to using wires involves the utilization of ceramic spheres, as demonstrated by Li et al.^28^. The desired number of spheres of known size can be strategically placed on the retina at specific positions. In their study, the smallest sphere had a diameter of 0.8 mm, which is clearly visible in both the OCT and the iiOCT imaging.

### Clinical applicability

Regarding the clinical applicability of our method, the time required for preoperative-intraoperative registration is a crucial factor for adoption. In clinical practice, diagnostic OCT and its screening are conducted preoperatively, which means the post-processing time is not critical. In contrast, tasks performed during surgery are time-sensitive and must be completed promptly to avoid disrupting the surgical workflow. The most time-consuming prerequisite for registration is the robotic acquisition of iiOCT data. This study evaluated various trajectories and subsampling methods, including a grid pattern, and a spiral configuration, supplemented by patches over the fovea and ONH. Prior to the peeling of the ERM, a vitrectomy is typically performed, which involves considerable movement within the eye. This step could be leveraged to obtain a random sparse scan of the retina’s structure. To improve our trajectories, it could be advantageous to capture smaller patches that concentrate on both the fovea and the ONH, as shown in Fig. 5. In contrast, the total acquisition time for T1, which ranges from approximately range0.5 min to 17 min depending on the spacing, is impractical. Consequently, it is essential to further decrease the point spacing and shorten the trajectory length.

In vivo registration precision will be affected by retinal movements due to respiration and heartbeat^31^. Cereda et al. found repetitive movement patterns with amplitudes of 10 $\upmu$m for heartbeat and 20 $\upmu$m for breathing^4^. While models can adjust for predictable movements^32^, unexpected non-periodic movements, like skipped heartbeats or snoring, remain challenging^33^. Furthermore, in vivo iiOCT sensing will be impacted by pathologies and disturbances such as bleeding and artifacts, necessitating sophisticated signal processing algorithms.

## Conclusion

This study introduced a pioneering pipeline for registering diagnostic OCT data with intraoperative iiOCT data, incorporating distortion correction of the OCT data by utilizing the geometrically precise iiOCT information. The proposed methods were rigorously validated using both an artificial model eye and an ex vivo porcine eye. The pipeline markedly improved the registration outcomes. Specifically, for the Lankenau model, the fiducial marker error was reduced to 103.0 $\upmu$m, and the Chamfer distance was decreased to 54.7 $\upmu$m. In the case of porcine eyes, while curvature correction did not improve the fiducial marker error, it did achieve an average reduction in Chamfer distance by 135 $\upmu$m. Further improvements could be realized through the adoption of more sophisticated validation techniques or the deployment of advanced hardware. This work represents a significant advancement in the realm of robot-assisted vitreoretinal surgery by seamlessly integrating diagnostic OCT with intraoperative iiOCT, thereby offering surgeons precise representations of the retina’s structure. This integration facilitates a suite of advanced robotic assistance functions, enhancing surgical precision and outcomes.

## Data Availability

The datasets generated during and/or analysed during the current study are available from the corresponding author on reasonable request.

## Code availability

The code is available from the corresponding author on reasonable request.
